# Fabrication and Characterization of Ce^3+^-Doped Lithium Alumino-Silicate Scintillating Glass–Ceramic and Fiber

**DOI:** 10.3390/ma17184481

**Published:** 2024-09-12

**Authors:** Yongya Wang, Fanbo Meng, Huiyu Chen, Wenqin Luo, Shunjian Xu, Chunyan Lv

**Affiliations:** 1 Huzhou Key Laboratory of Green Energy Materials and Battery Cascade Utilization, Huzhou College, Huzhou 313000, China; wangyongya@zjhzu.edu.cn (Y.W.);; 2 Key Laboratory of Materials for High Power Laser, Shanghai Institute of Optics and Fine Mechanics, Chinese Academy of Sciences, Shanghai 201800, China; 3 Department of Material Chemistry, Huzhou University, Huzhou 313000, China

**Keywords:** Ce^3+^ doping, lithium aluminum silicate scintillating glass, spectroscopic characteristics, glass–ceramic hybrid scintillating fiber

## Abstract

Ce^3+^-doped lithium alumino-silicate (Li-Al-Si) scintillating glass was prepared using a melting method and crystallized via heat treatment. X-ray diffraction and transmission electron microscopy confirmed the presence of nanocrystals in the materials. Radioluminescence spectra, obtained by X-ray excitation, and luminescence spectra, obtained by 338 nm excitation, showed that the luminescence intensity increased after crystallization. The glass was combined with pure silica as the inner cladding to fabricate a hybrid fiber core using a melt-in-tube technique. The composition of the fiber core was examined using an electron probe microanalyzer. The glass fiber produced strong blue luminescence under UV excitation. After a micro-crystallizing heat treatment of the hybrid fiber at 850 °C in a reducing atmosphere, a Ce^3+^-doped lithium alumino-silicate glass–ceramic scintillating hybrid fiber was obtained. The nanocrystal structure of the fiber core was examined using micro-Raman spectroscopy. Excitation and luminescence spectra of the hybrid fiber before and after micro-crystallization were measured using microspectrofluorimetry. The results demonstrated that the fiber remained luminous after micro-crystallization. Hence, this work provides a new way to prepare scintillating glass–ceramic hybrid fibers for neutron detection.

## 1. Introduction

As one of the key components in scintillator detector devices, scintillator materials play an important role in the development of new-generation scintillator detectors. Scintillating glass combines the advantages of low cost, easy fabrication of large pieces, suitability for mass production, and the capability to be drawn into optical fiber. Hence, extensive research has been carried out on this material by many researchers [1,2,3]. In particular, scintillating glass for neutron detection has long been a major research focus [4,5]. However, the light yield (LY) in glass scintillators is limited by the absence of long-range order in their atomic structure. As a result, the mean free path of excitons in glasses is very small and the delivery of electronic excitations to luminescent centers through the exciton migration has low efficiency. Therefore, effective scintillation in glasses can be achieved only through the direct excitation of luminescent centers, which include dopants such as Ce ions. Scintillating glass, represented by the Li-Al-Si glass system, has high detection efficiency and rapid response via the energy transfer from Li to Ce using the high-neutron-response characteristics of 6Li [6,7]. However, the synthesis of glass doping with a high content of cerium is still challenging due to its heterovalent properties. Although glass materials have unique advantages, their luminescence yield is often lower compared to single-crystal and ceramic scintillators. Glass–ceramic scintillators can maintain the advantages of easy preparation of glass materials while also possessing the high light yield characteristics of crystalline scintillators [8,9].

Scintillating fiber has the unique advantages of small size, a high spatial resolution, and great flexibility, showing promising applications in microbeam dosimetry, high-resolution imaging, and high-accuracy positioning for various radiation types, such as X-ray, γ-ray, or neutron radiation. For example, in boron neutron capture therapy, a single scintillating fiber can effectively provide the flux and intensity distribution of thermal neutrons in the cancer area in real time [10,11,12]. However, it is difficult to manufacture fibers from Li-Al-Si glass because of its easy crystallization during fiber drawing, which limits the production of fibers with good optical qualities and high efficiency in thermal neutron capture. Consequently, few studies have reported on Li-Al-Si glass scintillating fibers [13,14,15]. Moreover, a single-crystal scintillating fiber has the disadvantages of slow growth speed, uneven diameter, and a lack of cladding structure, resulting in rough surfaces and large scattering losses. Some promising methods to solve these problems are the micro-crystallization of scintillating glass or the use of scintillating crystal as the fiber core, combined with pure silica as the inner cladding, to fabricate hybrid fibers [16,17,18]. However, fibers made from silica cladding with a scintillating crystal core are often unable to crystallize because of the diffusion of silica from the cladding into the core during the drawing process [19,20,21,22,23]. Li ions are typical glass crystallization ions [24,25,26]. If the fiber can be drawn out of the Li-Al-Si glass preform and silica tube, then micro-crystallization of the Li-Al-Si glass fiber core can be achieved via heat treatment. This is a possible strategy to prepare silica-clad scintillating glass–ceramic fiber through micro-crystalline processing.

This study provides a new method for preparing scintillating glass–ceramic fiber for high-energy ray detection. Li-Al-Si glass was selected as the matrix and Ce^3+^ as the doping ion to prepare Ce^3+^-doped Li-Al-Si scintillating glass via a melting method. Then, using Ce^3+^-doped Li-Al-Si scintillating glass as the core and silica as the inner cladding, a hybrid fiber was fabricated by a melt-in-tube technique. After the micro-crystallizing heat treatment, the micro-crystallization of the fiber core was achieved, and the spectral properties of the hybrid fiber were characterized.

## 2. Materials and Methods

### 2.1. Preparation and Characterization of Glass–Ceramic 

According to the composition of 0.6Li_2_O-0.206Al_2_O_3_-1SiO_2_-0.006CeO_2_, SiO_2_, Li_2_O, Al_2_O_3_, and CeO_2_ with a total mass of 60 g were used as starting materials. The purity and the factories of raw materials are listed in Table 1. The well-mixed powders were placed into corundum crucibles and melted in a reducing atmosphere, using activated carbon powder as the reducing medium, at 1650 °C for 2 h. The use of a reducing atmosphere is seen because the precursor Ce is tetravalent, and this approach is capable of reducing it to the desirable trivalent form. Then, the melt was poured onto a preheated steel mold and transferred to a muffle furnace for annealing at 500 °C for 23 h.

**Figure 1:**
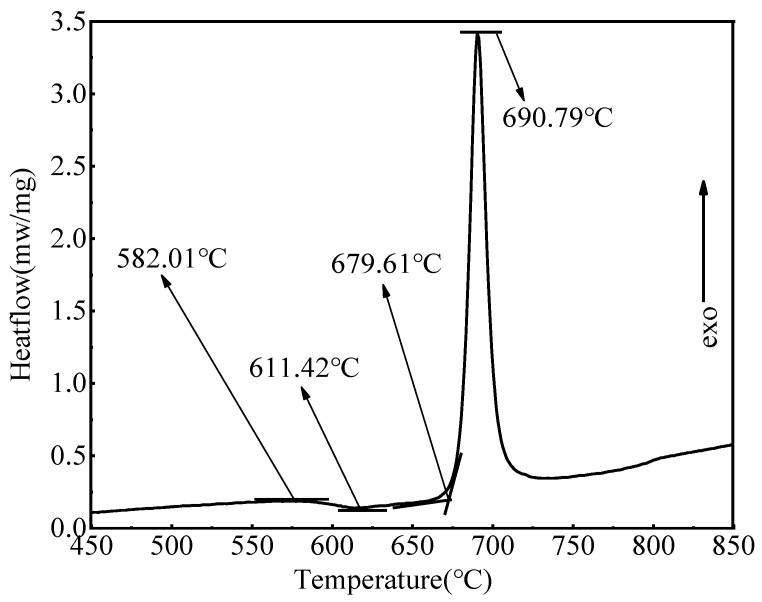
DSC curve of the glass powder.

A small part of the obtained basic glass was ground into powder to determine its characteristic temperatures using a STA449/C differential scanning calorimeter (DSC) (Netzsch, Selbu, Germany) at a heating rate of 10 K/min. The DSC curve of the prepared glass, shown in Figure 1, determined the crystallization treatment system of the sample. A portion of the prepared glass was cut into circular slices (diameter = 25 mm, thickness = 1.5 mm) with polished surfaces, which were then crystallized by heat treatment as follows. A circular slice was placed in a muffle furnace with activated carbon powder as the reducing medium, and the temperature was increased from 25 °C to 620 °C or 650 °C over a time interval of 600 min and held for 5–120 min. Then, it was decreased to 580 °C over 60 min and held for 30 min, and lastly, it was decreased to 25 °C over an interval of 600 min.

Transmission spectra of the glass samples before and after micro-crystallization were collected in the wavelength range of 200–800 nm using a Lambda 950 spectrophotometer (Perkin Elmer, Waltham, USA). X-ray diffraction (XRD) analyses before and after micro-crystallization of the glass samples were performed using a D8 Advance diffractometer equipped with a SolX energy dispersion detector (Bruker, Bilerica, Germany). Excitation spectra of the glass before and after micro-crystallization were measured using an FLSP920 time-resolved fluorescence spectrometer (Edinburgh, Livingston, UK). X-ray excited radioluminescence spectra were measured using an X-ray tube (Mo anode, 100 kV, 1 mA) in conjunction with a SBP300 fluorescence spectrometer (Zolix Instruments Co., Beijing, China). The morphology and composition profiles of the micro-crystals in the glass after micro-crystallization were determined using an Tecnai G2 F20 S-TWIN transmission electron microscope (TEM)(FEI, Portland, OR, USA).

### 2.2. Preparation and Characterization of Fiber

Another portion of the Ce^3+^-doped Li-Al-Si glass was processed into a rod (diameter = 3 mm, length = 5 mm) and placed inside a silica tube (inner diameter = 3 mm, outer diameter = 25 mm, length = 150 mm). Then, the silica tube was drawn into a hybrid fiber with a core diameter of approximately 15 μm at a temperature of about 2000 °C on a custom-built fiber draw tower. The cross section of the hybrid fiber was measured using a DMRXE (Leica, Solms, Germany) polarizing microscope. The luminescent property of the hybrid fiber (length = 5 m) was measured under the excitation of a 351 nm laser. A JXA-8230 (JEOL, Beijing, China) electron probe micro-analysis (EPMA) microanalyzer was used to observe the fiber core using secondary electrons and to determine the elemental distribution using wavelength-dispersive spectroscopy (WDS).

The hybrid fiber was micro-crystallized by heat treatment as follows. The fiber was placed in a muffle furnace with activated carbon powder as the reducing medium, and the temperature was increased from 25 °C to 600 °C over an interval of 3 h and held steady for 1 h. Then, it was increased from 600 °C to 650–950 °C over an interval of 0.5–2 h and held steady for 1 h. Next, it was decreased to 600 °C over an interval of 0.5–2 h and held steady for 0.5 h, and finally, it was decreased to 25 °C over an interval of 10 h. Raman spectra were collected in the wavelength range of 100–1600 nm before and after the micro-crystallization of the hybrid fiber core using a laser Raman microscope (Renishaw in Via, Gloucestershire, UK) equipped with a 785 nm laser. Each measurement was controlled at the same power, spot size (10 μm, with a 50× objective), and exposure time. Fluorescence spectra of the hybrid fiber core before and after micro-crystallization were measured using an Edinburgh FLSP920 time-resolved fluorescence spectrometer (Edinburgh, Livingston, UK). A section of the Ce^3+^-doped Li-Al-Si glass–ceramic scintillating hybrid fiber (length = 10 cm) was selected for use in testing the luminescent property under the excitation of a 351 nm laser.

## 3. Results and Discussion

### 3.1. Glass and Glass–Ceramics

**Figure 2:**
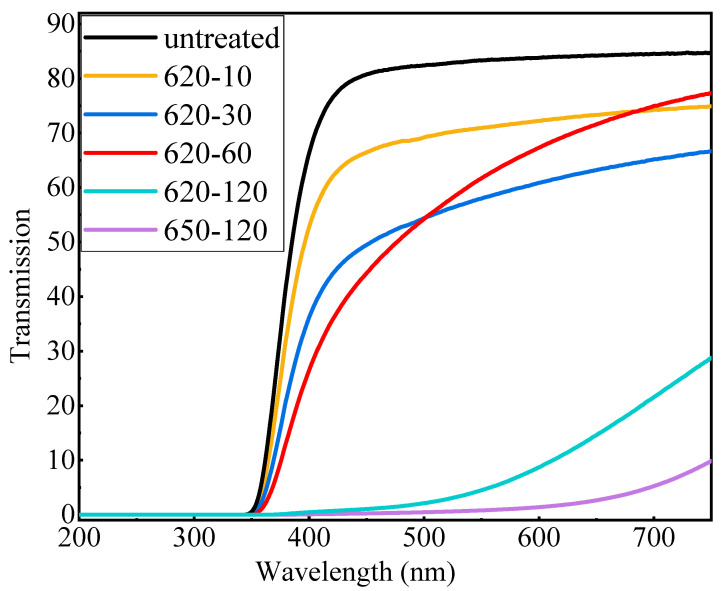
Transmission spectra of the glass with different treatments (the numbers in the first column of inset are heat treatment temperatures, and those in the second column are heat treatment times (min)).

The transmission spectra of glass and glass–ceramics from 200–700 nm are shown in Figure 2. With an increase in the duration of heat treatment at 620 °C, the transmittance of the glass decreased significantly, indicating that the glass began to crystallize. When the heat treatment time was increased to 2 h at 620 °C, the glass–ceramic became opaque, which can be attributed to the growth of grains and the increase in quantity. Furthermore, it became fully opaque when the heat treatment temperature increased to 650 °C.

**Figure 3:**
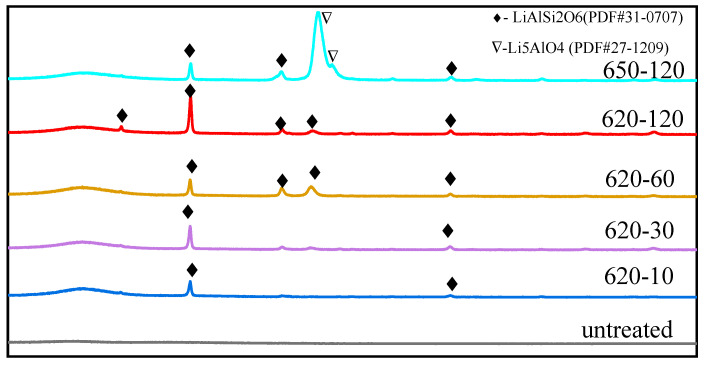
XRD patterns of glass samples with different treatments.

XRD patterns of the glass with different heat treatment conditions are shown in Figure 3. There were no micro-crystals in the untreated glass or the glass after heat treatment for only 5 min at 620 °C. As the heat treatment time was increased from 10 min to 120 min at 620 °C, a LiAlSi_2_O_6_(PDF#31-0707) crystal phase began to form. Moreover, as the heat treatment temperature was increased to 650 °C, new crystal phases precipitated from the sample. The variation in the intensity of diffraction peak of 650–120 could be due to the presence of Li_5_AlO_4_ (PDF#27-1209). This demonstrated that micro-crystals began to precipitate in the glass with an increase in heat treatment time or temperature.

**Figure 4:**
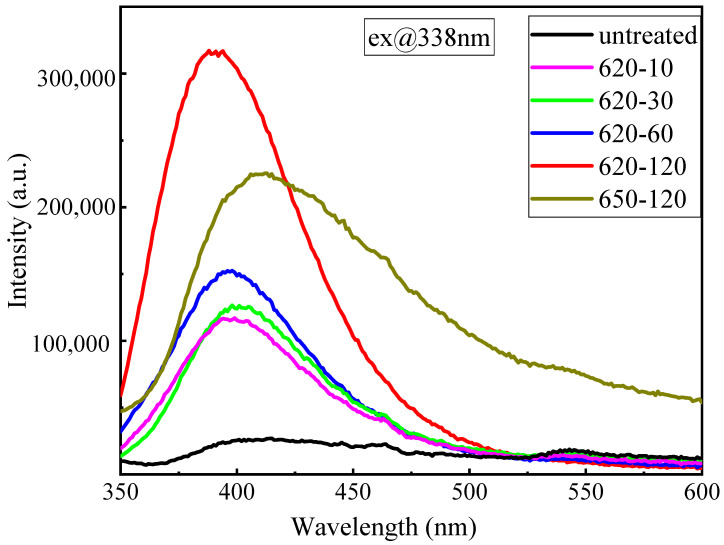
Emission spectra of the glass samples with different treatments.

The near-UV–visible luminescent spectra of glass and glass–ceramics under 338 nm UV irradiation are presented in Figure 4. The luminescent intensity increased with the heat treatment time at 620 °C. However, when the heat treatment temperature increased to 650 °C, the luminescent intensity weakened in comparison with that at 620 °C for the same heat treatment time. The reason was that the precipitation of other crystal phases and increased crystallinity reduce the transmittance of the sample treated by 650 °C.

**Figure 5:**
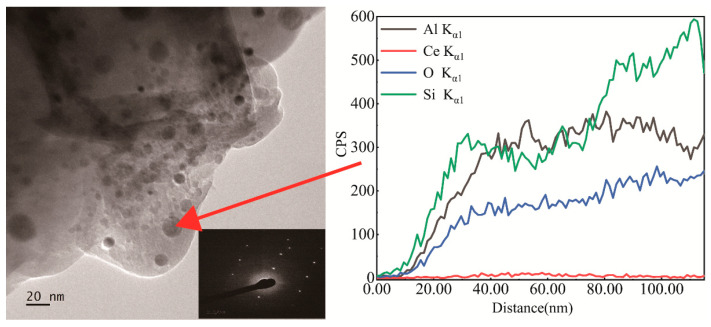
Transmission electron microscope photograph and composition of micro-crystals of glass after heat treatment at 620 °C.

To better understand the microstructure of glass–ceramics, they were analyzed by TEM. Figure 5 presents the TEM photograph and composition profile of micro-crystals in glass–ceramics after heat treatment at 620 °C for 120 min; the nanocrystal grain indicated by the red arrow was selected for an electron diffraction experiment. As shown in the inset photo of Figure 5, the grain crystallized. However, the composition profiles of the nanocrystal grain showed that Ce ions were not enriched within the grains, which was potentially due to the low concentration of cerium. Besides, the crystallinity of glass–ceramic samples further increases as the growth of micro-crystals progresses. Since there are no suitable lattice sites for Ce^3+^ ions to enter into LiAlSi_2_O_6_ crystals, the number of Ce^3+^ ions that can enter the micro-crystals is limited, resulting in a limited increase in fluorescence intensity after forming glass–ceramics.

**Figure 6:**
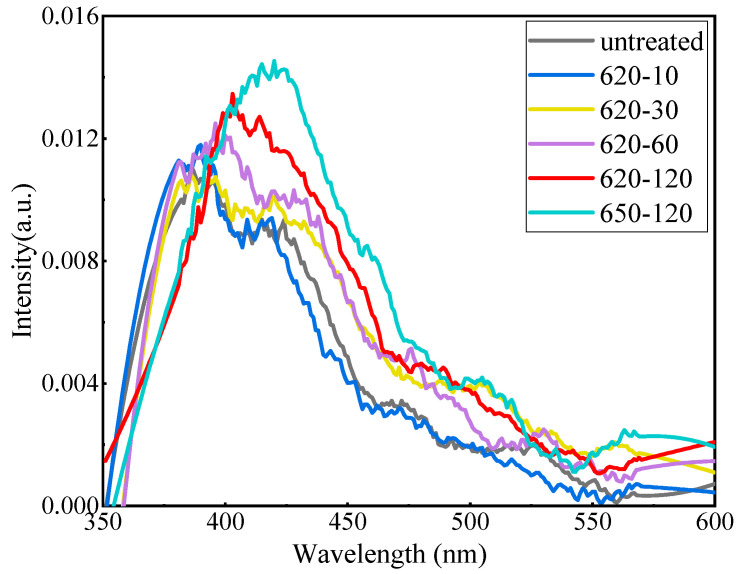
Radioluminescence spectra obtained by X-ray excitation of glass samples with different heat treatments.

The X-ray-excited radioluminescence spectra of the glass with different heat treatment conditions are given in Figure 6. The intensity increased with an increase in heat treatment time at 620 °C. The strongest fluorescence intensity occurred after heat treatment at 650 °C for 2 h. The fluorescence intensity of X-ray excitation is related to the relative content of glass and crystalline phases in the sample. The higher the content of crystalline phase, the stronger the fluorescence intensity. The 650–120 sample had a higher content of crystalline phase than other samples according to the XRD result, resulting in the highest overall fluorescence intensity.

### 3.2. Hybrid Fiber

**Figure 7:**
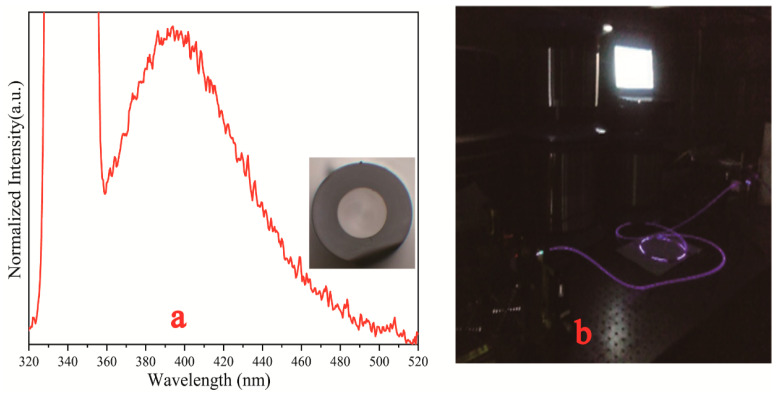
(**a**) Luminescence of hybrid fiber before crystallization treatment under excitation of 351 nm laser, and (**b**) photograph test site.

**Figure 8:**
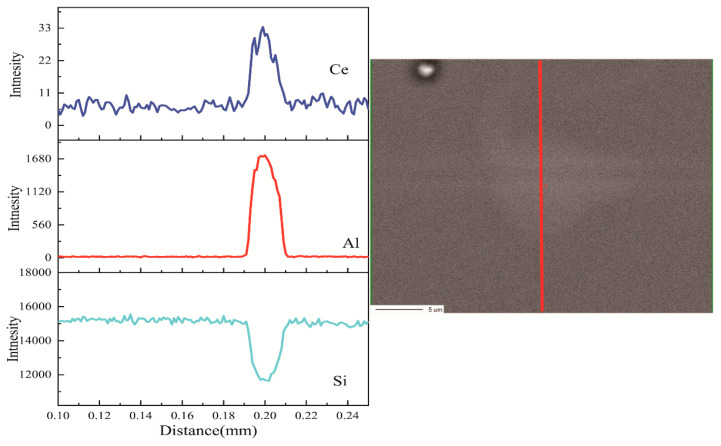
Scanning electron microscope photograph and composition profiles of fiber core obtained by EPMA.

Luminescence under excitation by a 351 nm laser is presented in Figure 7a, and the inset shows the cross section of the hybrid fiber. Figure 7b is a photograph taken during the measurement. As shown in the inset of Figure 7a, the interface between the fiber core and the silica cladding was well defined. This may have been due to the minimal diffusion of silica from cladding into the molten core during the fiber drawing process at 2000 °C. To determine whether the core maintained the composition of lithium aluminum silicon, the elemental distribution of the fiber was measured using EPMA, as shown in Figure 8.

The composition profiles in Figure 8 show that, although silica from the cladding diffused into the fiber core, and a considerable amount of aluminum and a small amount of cerium remained in the core, the amount of silica in the core was less than that in the cladding. In addition, the fiber still generated blue luminescence under the excitation of a near-ultraviolet laser, as shown in Figure 7a. These results showed that the core comprised Ce^3+^-doped lithium alumino-silicate glass and possessed scintillation luminescence properties, confirming that the hybrid fiber, with lithium alumino-silicate glass as the core and SiO_2_ glass as the inner cladding, was successfully prepared. Furthermore, the loss values of the core and inner cladding were 0.02 db/m@430–525 nm and approximately 1.0 db/m@351 nm (including the absorbed Ce^3+^ ions); the numerical aperture (NA) of the core was very small, and that of the inner cladding was 0.46. The fiber can exhibit good fluorescence effects with low loss values, indicating that this fiber has an excellent performance.

**Figure 9:**
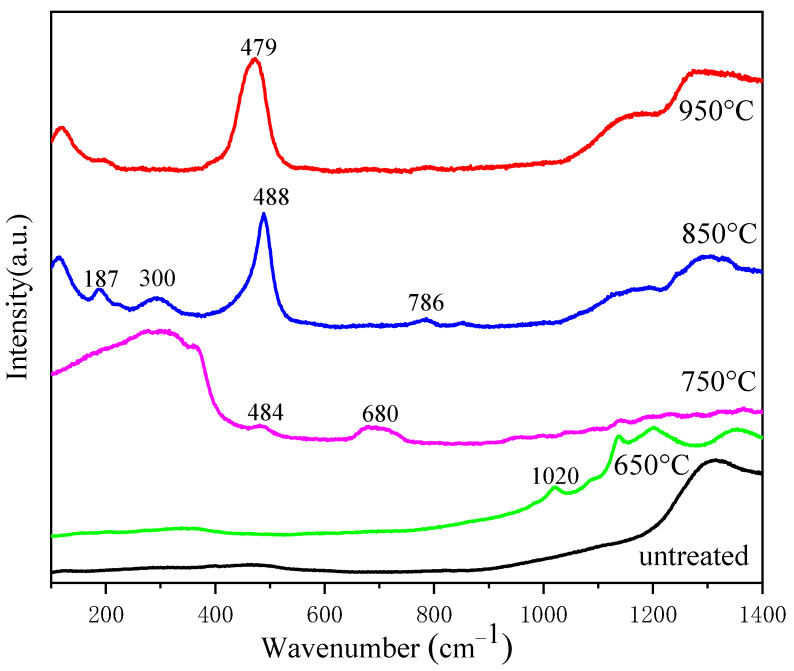
Micro-Raman spectra of the fiber core with different heat treatment temperatures.

**Figure 10:**
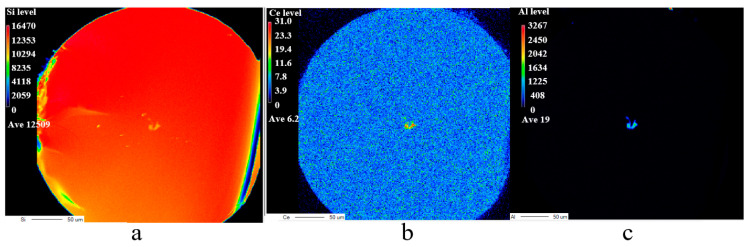
Composition distribution maps of hybrid fiber by EPMA after heat treatment at 850 °C. (**a**) distribution maps of Si, (**b**) distribution maps of Ce, (**c**) distribution maps of Al.

Next, micro-crystallization of the fiber core was performed to improve its luminous intensity; the fiber was used in the micro-crystallization experiment after removing its polymer cladding. Figure 9 displays the micro-Raman spectra of the fiber core before and after micro-crystallization, and Figure 10 presents the composition distribution map after micro-crystallization.

As seen from Figure 9, the Raman peak near 300 cm^−1^ is attributed to the alkali metal–oxygen bonding vibration, and the peaks appearing between 400 and 800 cm^−1^ are bending or stretching vibrations of bridging oxygen in silicon–oxygen tetrahedra. The peaks at 800–1200 cm^−1^ are symmetric stretching vibrations of non-bridging oxygen in silicon–oxygen tetrahedra [27,28].

The peak width of Si-O shear vibration around 480 cm^−1^ was significantly narrowed after micro-crystallizing heat treatment at 850 °C and 950 °C, indicating that the fiber gradually began to crystallize with an increase in temperature. Moreover, the micro-crystallizing temperature was very different from that of bulk glass because silica had diffused into the core. However, the excessively high temperature of micro-crystallizing heat treatment can induce the devitrification of the fiber core. Hence, a temperature of 850 or 950 °C was deemed suitable for micro-crystallizing heat treatment. Si, Al, and Ce distribution maps of the hybrid fiber faceplate after micro-crystallizing are shown in Figure 10, indicating that the amounts of Al and Ce in the fiber core were maintained at a certain level.

Rare earth ions are normally adopted as luminescence activators in scintillating glasses, with Ce^3+^ being the most common dopant because of its fast allowing of 5d→4f transition. At the same time, the luminescence of Ce^3+^ is strongly influenced by the chemical composition and atomic arrangement of the nearest neighbors [29,30]. The 5d energy level of Ce^3+^ is split into 5 components under an asymmetric crystal field [31]. With an increase in the doping concentration of Ce^3+^ in the glass matrix, the splitting width of the 5d level increased accordingly. As a result, the excitation band widened towards the low-energy end and the emission spectrum showed a significant redshift.

**Figure 11:**
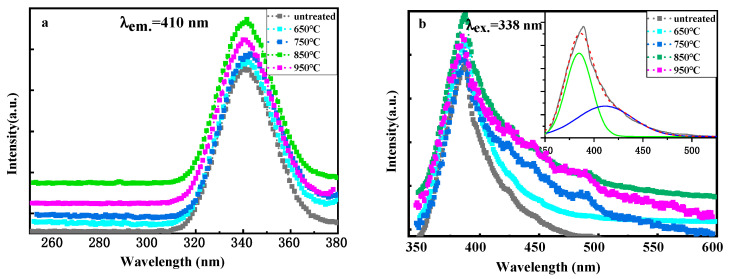
Micro-fluorescence spectra of scintillating fiber cores with different heat treatment temperatures. (**a**) excitation spectrum of scintillating fiber cores at 410 nm (**b**) emission spectrum of scintillating fiber cores at 338 nm.

**Figure 12:**
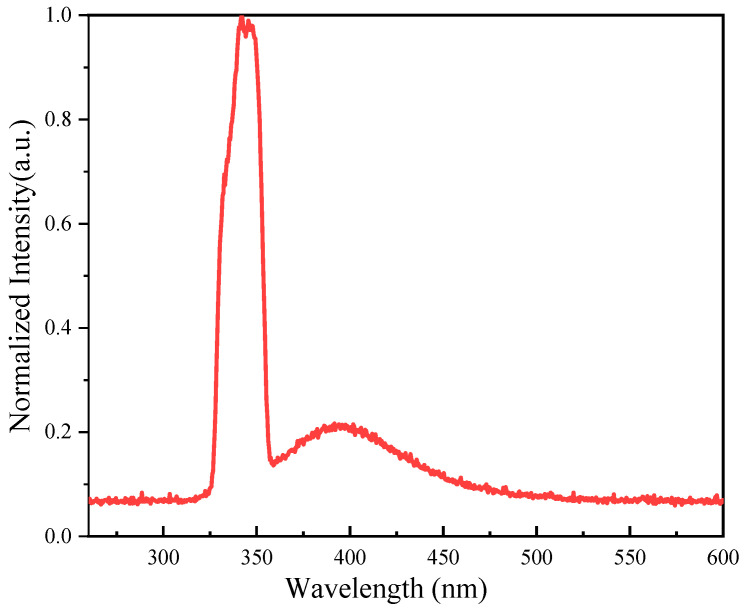
Fluorescence spectrum of glass–ceramic scintillating fiber under excitation by ultraviolet light at 340 nm.

The luminescent properties of the fiber before and after micro-crystallizing heat treatment were measured using micro-fluorescence, and the results are shown in Figure 11. Figure 11a shows the excitation spectra of the fibers after micro-crystallizing heat treatment at different temperatures by monitoring the emission line at 410 nm. Broad excitation bands at the wavelength region from 310 to 380 were detected in all samples, which corresponded to the transition from the ground ^2^F_5/2_ state to the 5d state of Ce^3+^ ions. The shapes of the excitation spectra remained nearly unchanged, indicating that the coordination environment of Ce^3+^ ions in all samples was similar. The emission spectra of the fibers under excitation at 338 nm are presented in Figure 11b. A broad emission band was observed from 350 to 600 nm, which had an asymmetric shape with a peak at 410 nm and a broad shoulder at longer wavelengths. The broad emission band can be deconvoluted into two Gaussian sub-peaks at 384.8 nm (25,986 cm^−1^) and 411.4 nm (24,307 cm^−1^), which can be ascribed to the electron transition from the lowest 5d orbit to the ^2^F_5/2_ and ^2^F_7/2_ levels of Ce^3+^. The energy gap between ^2^F_5/2_ and ^2^F_7/2_ was calculated to be 1679 cm^−1^, which is consistent with the theoretically calculated value (2000 cm^−1^) [32]. As revealed in Figure 11b, the emission tails at longer wavelengths were more evident after micro-crystallizing, which may be due to the occurrence of more prominent Raman scattering with the increase in the amount of nanocrystal phase precipitated from the glassy matrixes after heat treatment. The luminous intensity of the fiber subjected to micro-crystallizing heat treatment at 850 °C was slightly stronger than that of the fibers treated at other temperatures. Therefore, 850 °C was considered the optimum temperature for the micro-crystallizing heat treatment. A scintillating glass–ceramic hybrid fiber (length = 10 cm) subjected to micro-crystallizing heat treatment at 850 °C was fabricated, and its luminescence was measured as shown in Figure 12.

The excitation wavelength was 340 nm, emitted by an ultraviolet-visible spectrometer with a xenon lamp as the light source. As seen from Figure 12, the glass–ceramic scintillating fiber produced light with a center wavelength of 400 nm. This indicated that the fiber still functioned as a light guide after micro-crystallizing heat treatment, but the phenomenon of enhanced light emission was not observed. It is possible that most of the cerium ions were not precipitated along with the micro-crystals and that the concentration of cerium ions was diluted by the SiO_2_ molecules diffused into the fiber core. This work presented an effective method for the preparation of micro-crystalline fibers containing lithium ions. The next step is to improve the microstructure and to ensure that the cerium ions are precipitated along with the micro-crystals.

## 4. Conclusions

In this work, a novel Ce^3+^-doped Li-Al-Si glass was prepared using a melting method, and its scintillation performance was confirmed by its radioluminescence. The glass was crystallized into transparent glass–ceramics, as confirmed by XRD and TEM. Next, a scintillating glass fiber was fabricated using a melt-in-tube technique with Ce^3+^-doped Li-Al-Si glass as the core and silica as the inner cladding. EPMA results showed that the fiber core retained the composition of Ce^3+^-doped Li-Al-Si. Furthermore, when the glass fiber was subjected to micro-crystallizing heat treatment at 850 °C in a reducing atmosphere, a Ce^3+^-doped Li-Al-Si glass–ceramic scintillating hybrid fiber with good excitation spectrum properties was obtained. Micro-Raman measurements confirmed that the fiber core was micro-crystallized. The full width at half maximum (FWHM) of the fluorescence spectrum of the micro-crystalline fiber was narrowed, and the coordination field of cerium ions was changed after the micro-crystallizing heat treatment. Although the micro-crystalline fiber remained luminescent under excitation by ultraviolet light at 340 nm, the luminescence efficiency needs to be further improved. This may be achieved by improving the composition of the fiber core to ensure that cerium ions are precipitated along with the nanocrystals. The testing of scintillation efficiency requires the fabrication of optical fibers into array devices, which was a key focus of subsequent work. Hence, this work demonstrated that the micro-crystallization of the glass fiber core can be easily achieved by using a glass composition containing lithium, which provides a new approach for the fabrication of micro-crystalline fibers.

## Figures and Tables

**Table 1:** The purity and the factories of raw materials.

Raw Materials	Purity	Factory
SiO_2_	99.99%	Aladdin (Shanghai, China)
Li_2_O	98%	Aladdin (Shanghai, China)
CeO_2_	99.95%	Alfa Aesar (Shanghai, China)
Al_2_O_3_	99%	Aladdin (Shanghai, China)

## Data Availability

The original contributions presented in the study are included in the article, further inquiries can be directed to the corresponding author (due to privacy).
